# Reliability, validity, and feasibility of a method for assessing sport-specific reactive agility in badminton players

**DOI:** 10.7717/peerj.20972

**Published:** 2026-03-23

**Authors:** Jiachi Ye, Rui Cheng, Jingjie Zhou, Binghong Gao

**Affiliations:** 1 The Sport Science Research Institute, Nanjing Sport Institute, Nanjing, Jiangsu, China; 2 College of Athletic Performance, Shanghai University of Sport, Shanghai, China; 3 Faculty of Health Sciences and Sports, Ryerson Polytechnic University, Macao, China

**Keywords:** Agility, CODs, Decision-making, Performance, Testing

## Abstract

**Background:**

Reactive agility is a critical determinant of badminton performance, reflecting an athlete’s ability to rapidly perceive, decide, and execute movement responses under dynamic conditions. However, there is a lack of standardized and sport-specific assessment tools to evaluate this multidimensional skill in badminton. This study aimed to develop and evaluate the reliability, validity, and feasibility of a badminton-specific reactive agility test (B-RAT).

**Methods:**

Nine technical and three strength coaches assessed the content validity of the B-RAT using a four-point scale. A total of 201 professional athletes (119 male, 82 female) from provincial and national teams participated in the testing. Test-retest reliability was examined in 58 athletes across two sessions separated by seven days. Criterion validity was assessed in 28 single players by correlating B-RAT performance with simulated match outcomes, while construct validity was evaluated by comparing B-RAT results with established change of direction speed (CODS) tests and among athletes of different competition levels. Feasibility was assessed based on experts’ ratings of practicality, clarity, and resource requirements.

**Results:**

The B-RAT demonstrated excellent test-retest reliability (ICC = 0.90, CV = 5.82%), strong content validity (S-CVI = 0.93), and correlations with match ranking (*r* = 0.65 − 0.76, *p* < 0.05). In addition, B-RAT was significantly associated with five conventional CODS tests (*r* = 0.52 − 0.71, *p* < 0.01), and effectively differentiated athletes by competition level (*d* = 1.39 − 1.67; *η*^2^ = 0.29 − 0.43, *p* < 0.05). The feasibility score exceeded the required threshold (≥ 35/50).

**Conclusions:**

The B-RAT is a reliable, valid, and feasible test for assessing sport-specific reactive agility in badminton players. It effectively integrates perceptual-cognitive and motor components, providing a practical and standardized tool for evaluating performance, guiding individualized training, and monitoring agility development in competitive badminton contexts.

## Introduction

Agility refers to the rapid whole-body movement with change of velocity or direction in response to a stimulus (Paul, Gabbett & Nassis, 2016). Badminton is a high-intensity, intermittent racquet sport in which each rally is highly variable due to unpredictable match situations (Phomsoupha & Laffaye, 2015). Athletes must quickly execute complex movements, including change of direction, jumps, and strokes, in response to shuttle trajectory, tactical demands, and rapidly changing competitive conditions. Agility therefore plays a critical role in both defensive and counterattack performance, particularly during fast-paced rallies and sudden opponent attacks. Notably, male athletes in the National Badminton League show a strong negative correlation between agility and match performance (*r* =  − 0.83) (Tiwari, Rai & Srinet, 2011). This finding indicates that agility is a critical determinant of success in high-level badminton. Therefore, scientific agility assessment is essential in comprehensive athlete testing protocols.

Agility assessments are typically divided into change of direction speed (CODs) and reactive agility (RA) tests. Previous studies have used closed-skill tasks, including hexagon jumps (Fernandez-Fernandez et al., 2022), 505 CODs (Loturco et al., 2022), *T*-test CODs and SEMO CODs (Guo et al., 2021), on-court CODs (Fernandez-Fernandez et al., 2022), sideways CODs (Ooi et al., 2009), and four-conner CODs (Deng et al., 2025) to evaluate badminton-specific agility. However, CODs tests inadequately reflect athletes’ responsiveness to unpredictable stimuli in open environments, limiting their ecological validity (Jansen et al., 2021). RA tests, which incorporate random stimuli and decision-making components, better simulate competitive conditions and offer higher sport specificity and ecological validity, representing a key advance in scientific agility assessment (Paul, Gabbett & Nassis, 2016; Ye et al., 2025).

A sport-specific RA test for badminton should integrate multiple dimensions, including cognitive component, sport-specific technique, test distance, number and angles of CODs, task representativeness, and court conditions (Jansen et al., 2021). Badminton athletes frequently accelerate, decelerate, and change of direction within short periods, demanding not only physical fitness but also high perceptual and reactive skills. Efficient execution of sport-specific techniques during rapid directional changes is a key determinant of match outcomes and a discriminator of athletic performance level (Sekulic et al., 2019). Moreover, athletes perform a stroke approximately every 2 s, continuously engaging in deceleration and re-acceleration throughout a rally (6–12 s), requiring rapid court coverage and precise stroke execution (Phomsoupha & Laffaye, 2015). Therefore, the badminton-specific RA test (B-RAT) must integrate physical and cognitive demands to accurately reflect match-relevant agility.

Recently, several RA tests based on photic or visual stimuli have been developed to replicate match-specific movement patterns and energy demands (Robertson et al., 2017; Robertson, Burnett & Cochrane, 2014). While these tests partially simulate real-game scenarios, their reliability, validity, and feasibility have not been systematically verified. High equipment costs and operational complexity further limit their widespread use. Therefore, this study aims to develop a scientifically rigorous, reliable, and feasible B-RAT and systematically evaluate its test-retest reliability, content validity, correlations with match performance and existing CODs tests, and practical feasibility. We hypothesize that B-RAT will demonstrate excellent reliability, effectively differentiate athletes of varying competitive levels, show significant correlations with match performance and existing CODs tests, and possess high feasibility for implementation in training and research settings.

## Materials and Methods

### Participants

To establish content validity and feasibility, nine technical coaches and three strength and conditioning coaches were invited to evaluate the newly designed B-RAT. The coaches averaged 41.11 ± 9.56 years of age and 14.50 ± 9.72 years of coaching experience, all of whom had over five years of experience on provincial teams. The athletes sample size was estimated separately for different study purposes: test-retest reliability (intraclass correlation coefficient set at 0.5–0.8, requiring 22 subjects), convergent validity (effect size set at 0.3–0.7, requiring 28 subjects), and discriminant validity (based on preliminary data of three groups, requiring 39 subjects). Ultimately, 201 badminton athletes were recruited using convenience sampling (female, *n* = 82; age: 15.84 ± 2.58 years; height: 167.47 ± 5.52 cm; body mass: 59.62 ±7.73 kg; male, *n* = 119; age: 16.75 ± 2.65 years; height: 176.64 ± 7.60 cm; body mass: 64.45 ± 9.40 kg). Inclusion criteria were current national or provincial team membership, at least five years of systematic training, a minimum weekly training volume of 20 h, and regular participation in national-level competitions within the past year. Athletes with musculoskeletal injuries or other medical conditions that could impair testing were excluded. All participants and their legal guardians provided written informed consent prior to participation. This study was approved by the Nanjing Sport Institute Human Research Ethics Committee (No. RT-2024-26).

### Study design

This study aimed to evaluate the reliability, validity, and feasibility of the B-RAT. Content validity was confirmed by 12 badminton experts, and feasibility was assessed using a 10-item questionnaire rated by the same panel. A total of 58 athletes (30 males, 28 females) completed the B-RAT on two occasions separated by 7 days to assess test–retest reliability. Criterion validity was determined by comparing B-RAT performance with simulated round-robin match results in 28 singles players. Convergent validity was examined in 34 athletes, who completed the B-RAT followed sequentially by the traditional 505 CODs, modified *T*-test CODs, SEMO CODs test, sideways CODs, and four-conner CODs test on the same day. Discriminant validity was evaluated by comparing B-RAT performance across competitive levels. The 202 participants were classified as elite, highly trained, or trained according to McKay et al. (2022).

Following the recommendations of Currell & Jeukendrup (2008) for athletic performance testing, the procedures in the present study were standardized as follows: all tests were conducted in an indoor badminton hall between 14:00 and 18:00; participants refrained from vigorous exercise for 24 h prior to testing and performed two familiarization trials before each test to ensure familiarity with the procedures and stimulus–response requirements; a 15-minute standardized warm-up was performed before testing, including running, dynamic stretching, and progressively intensified jumping and CODs drills; each test was performed twice with a 1-minute rest interval, and the mean of the two trials was used for analysis; a 5-minute interval was provided between different tests for equipment adjustments and instructions for subsequent tests; verbal encouragement and immediate feedback were provided throughout to optimize performance.

### Parameters

#### Content validity assessment

To evaluate the content validity and feasibility of the B-RAT, an expert review method was employed. The content validity assessment form was developed based on the sport-specific agility test evaluation scale proposed by Jansen et al. (2021) (Appendix I) and completed by a panel of 12 badminton experts. Experts reviewed the definition of agility, a B-RAT demonstration video, its cognitive component, sport-specific technique, test distance, number and angles of CODs, task representativeness, and court conditions. Each item was then rated for relevance and applicability to in-game agility using a 4-point scale, with 1 = not relevant, 2 = somewhat relevant, 3 = quite relevant, and 4 = highly relevant (Davis, 1992).

#### Feasibility assessment

The feasibility of the B-RAT was evaluated using a 10-item checklist adapted from previous studies (Chiwaridzo et al., 2018; Robertson et al., 2017) (Appendix II). Items were categorized into three levels of priority. High-priority items included equipment needed (a), procedure of the test (b), possible modifications to the test or equipment (c), and cost analysis (d). Medium-priority items consisted of average duration (e), human resources required (f), and scoring and interpretation of test scores (g). Low-priority items included age-specificity (h), logical acceptability (i), and safety (j). Experts viewed the B-RAT demonstration video and rated each item on a 3-point scale (0 = not feasible, 1 = somewhat feasible, 2 = feasible). The overall feasibility score was calculated using the following weighted formula: total score = 4a + 4b + 4c + 4d + 2e + 2f + 2g + 1 h + 1i + 1j, yielding a maximum of 50 points. A threshold of ≥35 points was adopted to indicate high feasibility (Chiwaridzo et al., 2018).

#### Badminton-specific reactive agility test

**Figure 1:**
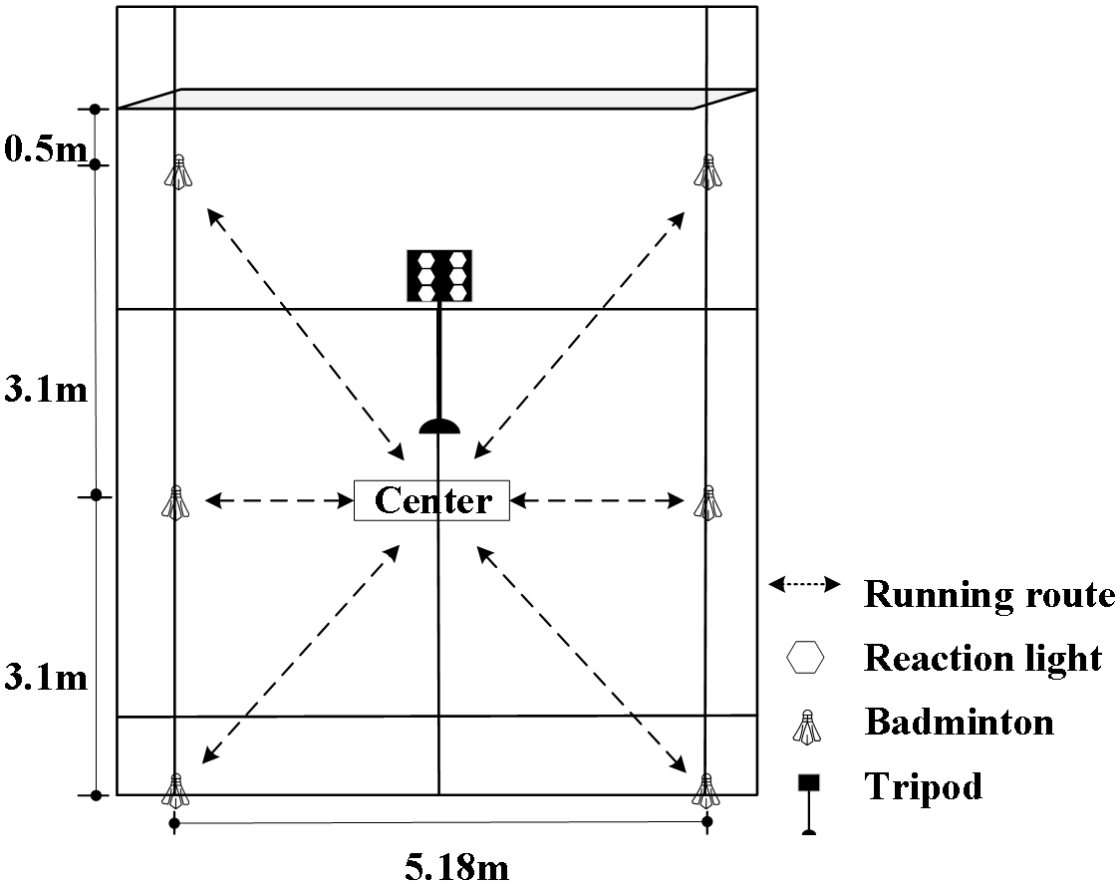
Schematic diagram of badminton-specific reactive agility test.

The B-RAT assessed sport-specific agility in badminton players using the timing system (Reaction X, China). The testing area consisted of six shuttlecock targets arranged in a 6.2 m × 5.18 m rectangle, with six reaction lights mounted on a stand 0.5 m in front of the center and connected *via* Bluetooth to an iPad. Players stood with feet parallel at the center and, upon random light activation, sprinted to the target to touch the shuttlecock and then returned to touch the light. Each athlete received a unique randomized sequence of light activation to minimize order effects and potential learning bias. The test ended after all six targets were completed, with a total running distance of approximately 21.3 m. Players used badminton-specific footwork and their racquet hand to touch the targets, while the software recorded segment and total times. Tests were repeated if players had incorrect stance, ran to the wrong target, missed a target, or used the non-racquet hand (Fig. 1).

#### Change of direction speed tests

*505 CODs test.* The 505 CODs test was performed using a timing gate system (Fusion Sport, Australia). Players sprinted from the starting line to a marker at 15 m, executed a 180° turn, and returned to the 10 m gate, covering a total distance of 10 m (Freitas et al., 2023).

*Modified T*-*test CODs.* The modified *T*-test CODs was conducted on a badminton court with cones positioned at designated locations; players performed a sequence of forward sprints, lateral shuffles, and backpedaling over approximately 19.4 m while facing the net throughout.

*SEMO CODs test.* The SEMO CODs test was conducted in a 5.7  × 5.1 m area, where players completed a sequence of lateral shuffles, backpedaling, and sprints, with a total running distance of ∼37 m (Guo et al., 2021).

*Sideways CODs test.* The sideways CODs test was performed using the Reaction X system (Reaction X, China); players moved laterally between singles sidelines to strike shuttlecock targets 10 times, covering ∼25.5 m (Ooi et al., 2009).

**Figure 2:**
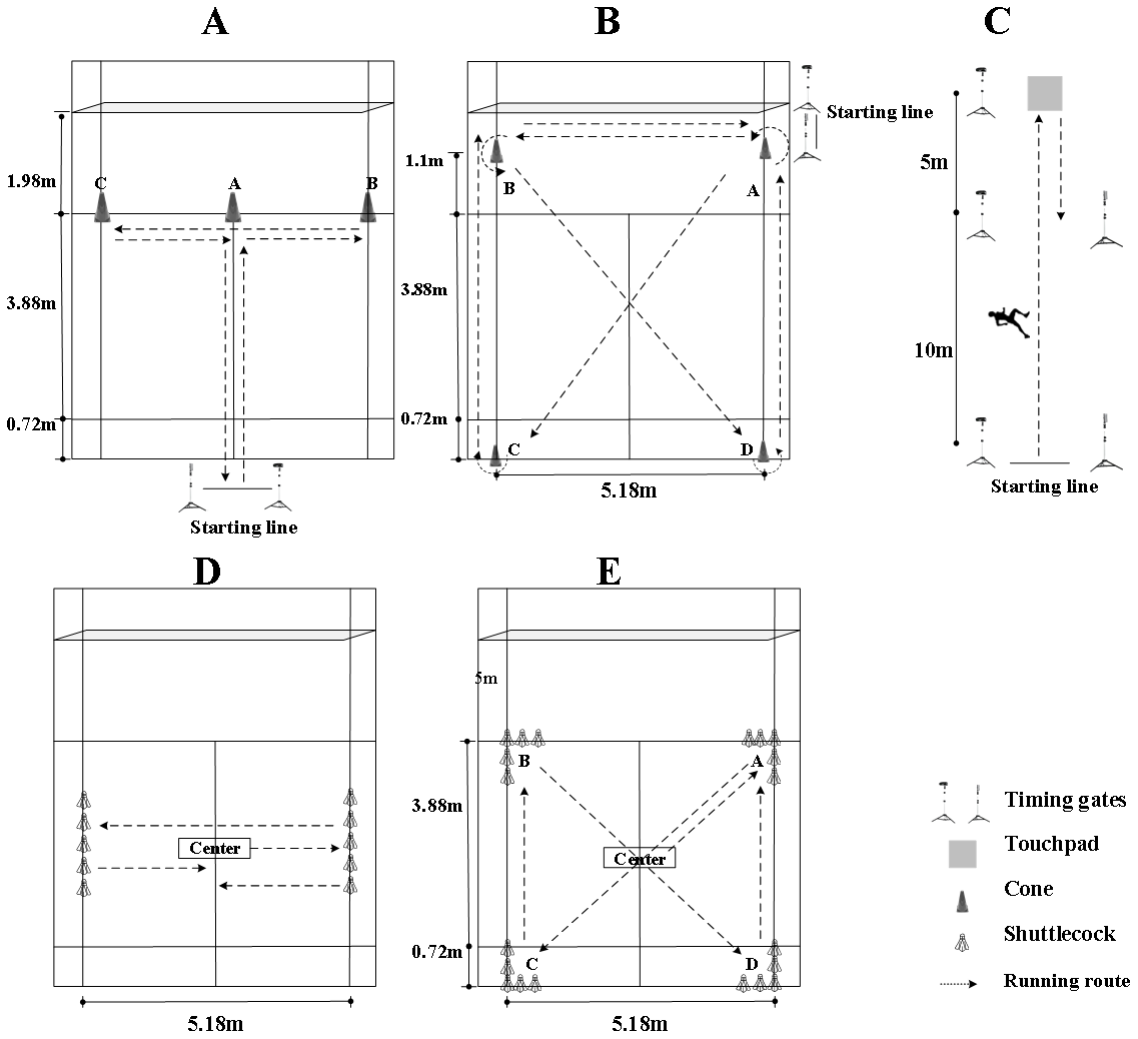
A schema for five change of direction speed (CODs) tests: *T*-test CODs (A), SEMO CODs (B); 505 CODs (C); sideways CODs (D); four-conner CODs (E).

*Four-corner CODs test.* The four-conner CODs test was conducted in a 4.6  × 5.1 m area, where players moved rapidly to four shuttlecock targets in sequence and repeated the pattern 20 times, for a total distance of ∼114 m (Fig. 2).

#### Match performance assessment

Following the completion of the physical fitness tests, all participants resumed their routine training programs without additional intervention. Within one month, they engaged in an internal simulated competition designed to replicate official match conditions. The competition adopted a round-robin format, in which each player faced every other participant once. Matches were conducted under standard badminton rules, ensuring consistency in scoring and officiating. Upon completion of all fixtures, players were ranked based on the number of matches won, with lower rank numbers indicating better performance. In the event of a tie, rankings were further determined by game differential and, if necessary, point differential. This competitive setting provided an objective measure of match performance, serving as a practical indicator of athletes’ technical-tactical effectiveness and competitive outcomes.

### Statistical analyses

All statistical analyses were performed using IBM SPSS for Windows (version 24.0; SPSS, Inc., Armonk, NY, USA). Continuous variables are presented as mean ± SD. Relative reliability of the B-RAT was evaluated using a two-way mixed-effects ICC (2,1), with ICC > 0.90 considered excellent, 0.75–0.90 good, 0.50–0.75 moderate, and < 0.50 poor (Koo & Li, 2016). CV < 10% was considered acceptable. Absolute reliability was examined using standard error of measurement and minimal detectable change. Content validity was assessed at the Item-level Content Validity Index (I-CVI) and Scale-level Content Validity Index (S-CVI) levels, with I-CVI > 0.78 and S-CVI > 0.90 indicating acceptable validity (Lynn, 1986). Correlations between B-RAT, match performance, and other agility tests were analyzed using Pearson or Spearman coefficients depending on data normality (small: 0.1 ≤*r* < 0.3; moderate: 0.3 ≤*r* < 0.5; large: 0.5 ≤*r* < 0.7; very large: 0.7 ≤*r* < 0.9; near perfect: *r* ≥ 0.9; perfect: *r* = 1)) (Hopkins et al., 2009). Differences in B-RAT performance between match outcome groups were tested using independent samples t-tests or Mann–Whitney U tests, with Cohen’s d effect size reported (trivial: *d* < 0.20; small: 0.20 ≤*d* ≤ 0.50; medium: 0.50 <*d* ≤ 0.80; large: *d* > 0.80) (Bakeman, 2005). One-way ANOVA compared B-RAT performance across competitive levels, using LSD *post hoc* tests when variances were equal and Welch ANOVA with Tamhane T2 *post hoc* tests when unequal. Eta-squared (*η*^2^) was used to report effect sizes for group comparisons (small: 0.010–0.059; medium: 0.059–0.138; large: >0.138) (Cohen, 2013). A two-tailed *p*-value < 0.05 was considered statistically significant.

## Results

Test re-test reliability data are presented in Table 1. The overall ICC was 0.90 (95% CI [0.84–0.94]), and ICCs for male and female athletes were 0.79 (95% CI [0.58–0.89]) and 0.84 (95% CI [0.69–0.92]), respectively. CVs were below 6% across all groups, and SEM and MDC95 ranged from 0.31 to 0.34 s and 0.86 to 0.94 s.

**Figure 3:**
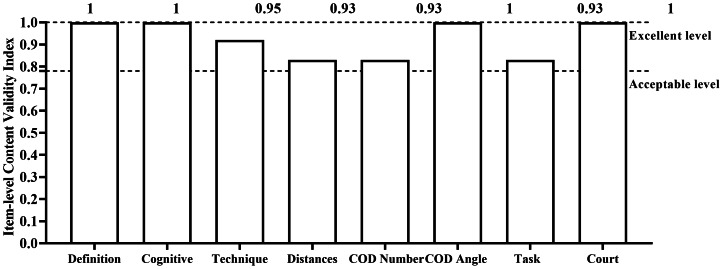
Content validity of the badminton-specific reactive agility test.

Content validity analysis indicated I-CVI values of 1 for definition, sport-specific cognition component, angles of CODs, and court condition, with 0.92 for sport-specific technique, and 0.83 for test distance, number of CODs, and task representativeness. The overall S-CVI was 0.93 (Fig. 3).

**Figure 4:**
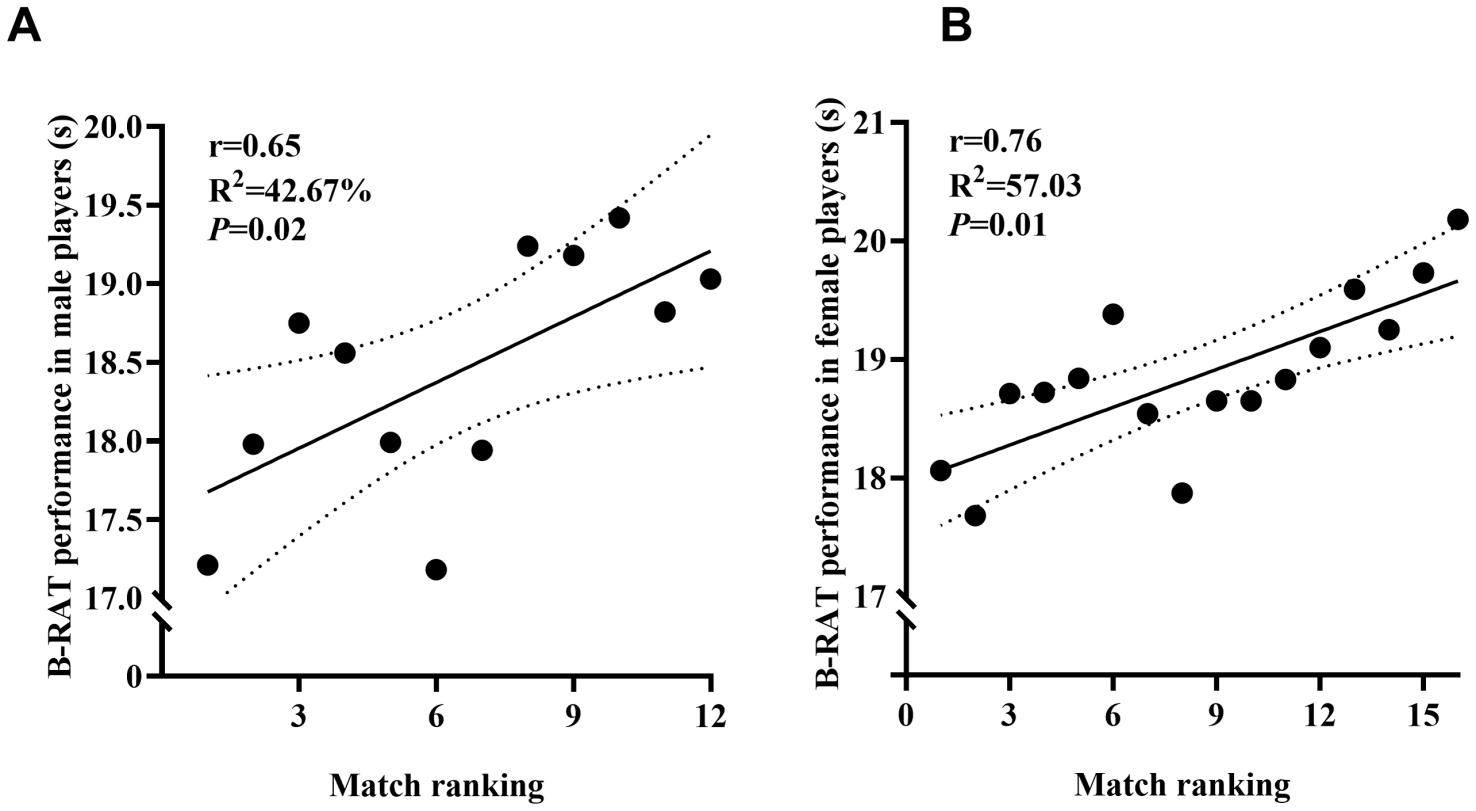
The relationship between badminton-specific reactive agility and simulated match performance.

**Figure 5:**
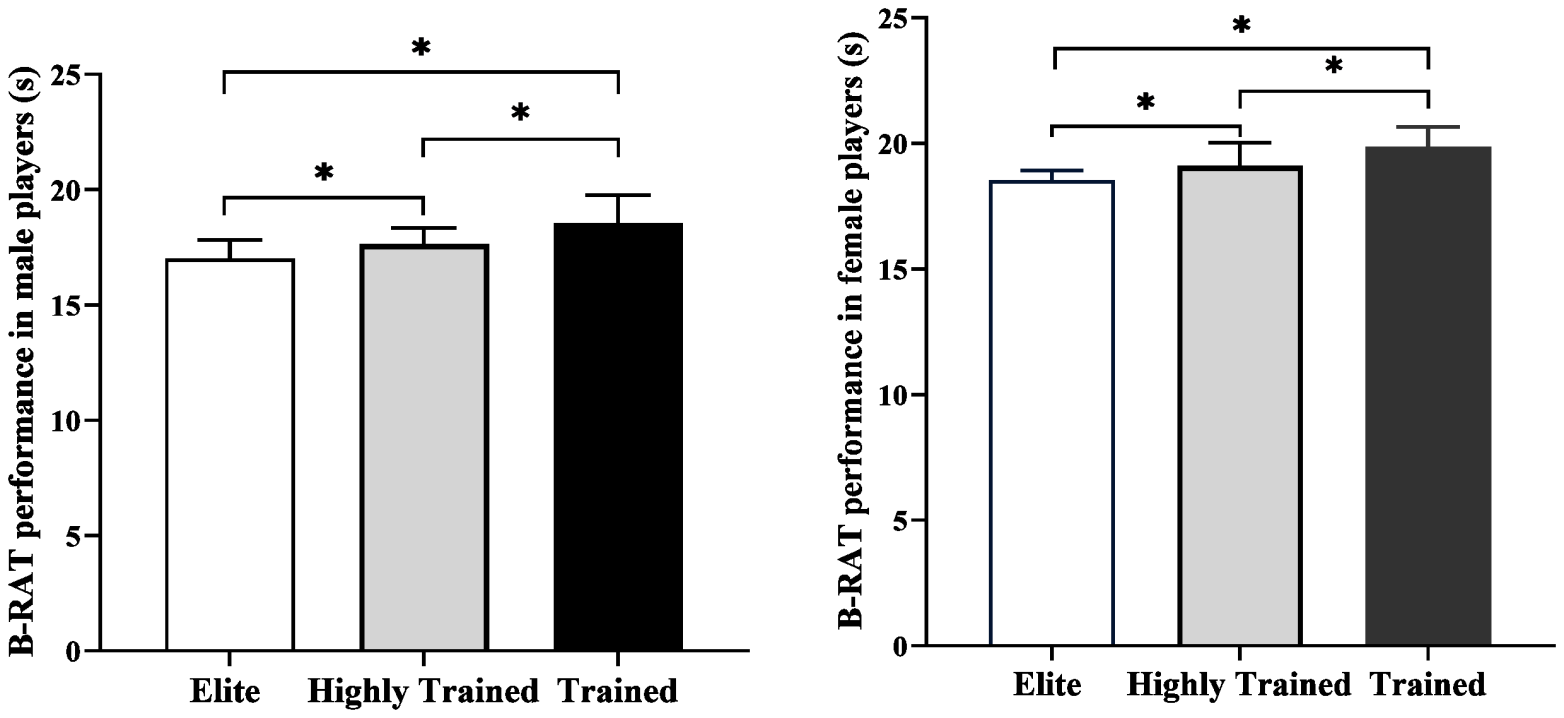
Differences in B-RAT performance across competitive levels. Note B-RAT, badminton-specific reactive agility test; * significant *p* < 0.05.

B-RAT scores were strongly correlated with simulated match performance (male *r* = 0.65; female *r* = 0.76; *p* < 0.05) (Fig. 4). Significant differences were observed across competitive levels. Male elite athletes were faster than highly trained and trained athletes (difference: 0.65–1.56 s, *p* < 0.05, *η*^2^ = 0.29), and female elite athletes were faster than highly trained and trained athletes (difference: 0.58–1.32 s, *p* < 0.05, *η*^2^ = 0.43) (Fig. 5).

**Table 1:** Test-retest reliability of the badminton-specific reactive agility test.

Participants	Test 1 (s)	Test 2 (s)	ICC (95% CI)	CV (%)	SEM (s)	MDC_95_ (s)
Overall (*n* = 58)	18.21 ± 0.96	18.12 ± 1.11	0.90 (0.84–0.94)	5.82	0.33	0.91
Male (*n* = 30)	17.55 ± 0.72	17.35 ± 0.73	0.79 (0.58–0.89)	4.19	0.34	0.94
Female (*n* = 28)	18.92 ± 0.72	18.95 ± 0.82	0.84 (0.69–0.92)	4.10	0.31	0.86

**Notes.**

ICC intraclass correlation coefficient CV coefficient of variation SEM standard error of measurement MDC_95_ minimal detectable change with 95% confidence intervals

B-RAT was strongly correlated with the modified *T*-test CODs (*r* = 0.64, *p* < 0.001), SEMO CODs test (*r* = 0.58, *p* < 0.001), and sideways CODs test (*r* = 0.56, *p* = 0.01), and moderately correlated with the four-conner CODs test and 505 CODs test (*r* = 0.48−0.50, *p* = 0.01) (Table 2).

**Table 2:** Correlations between B-RAT and other change of direction speed tests.

Test Type	Result (s)	r (95% CI)	*p*	R^2^	Adjusted R^2^
SEMO CODs	11.49 ± 0.11	0.58 (0.28–0.76)	<0.001	0.34	0.32
Modified *T*-test CODs	5.81 ± 0.09	0.64 (0.40–0.81)	<0.001	0.40	0.38
Sideways CODs	15.56 ± 0.17	0.56 (0.25–0.79)	0.01	0.31	0.29
Four-conner CODs	32.44 ± 0.24	0.48 (0.19–0.72)	0.01	0.23	0.20
505 CODs -left	2.45 ± 0.02	0.50 (0.25–0.71)	0.01	0.25	0.22
505 CODs -right	2.42 ± 0.02	0.50 (0.29–0.69)	0.01	0.25	0.22

**Figure 6:**
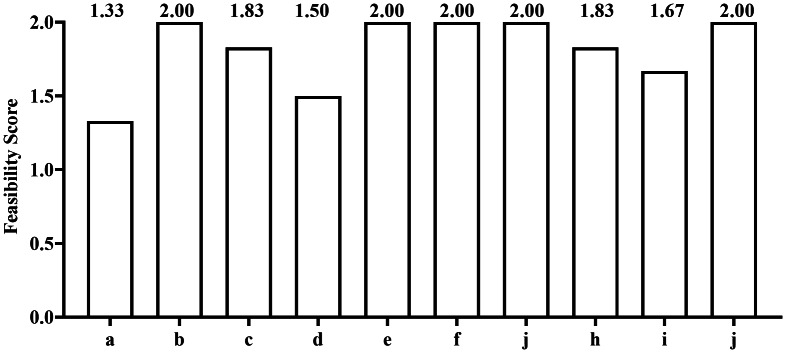
Feasibility of the badminton-specific reactive agility test. Note: (A) equipment needed; (B) test procedure; (C) possible modifications; (D) equipment cost; (E) test duration; (F) personnel required; (G) scoring and interpretation; (H) age-specificity; (I) logical acceptability; (J) safety.

The feasibility assessment demonstrated that B-RAT scored well across all priority levels, with an average total score of 44.17, substantially exceeding the feasibility threshold of 35 points (Fig. 6).

## Discussion

This study aimed to develop and evaluate a new B-RAT and to examine its reliability, validity, and feasibility in badminton players. The results demonstrated that the B-RAT shows good relative and absolute reliability. Furthermore, strong evidence of validity was observed, including content validity, criterion validity and known-groups discriminative validity. Moderate correlations were also identified between B-RAT performance and commonly used CODs tests. Finally, a feasibility score of 44.17 indicates that the B-RAT has high practical applicability in applied sport settings.

### Reliability

Reliability refers to the reproducibility of values of a test, assay or other measurement in repeated trials on the same individuals (Hopkins, 2000). Reliability is typically assessed as relative or absolute. Relative reliability evaluates the stability of individual rank order across repeated trials, commonly quantified using ICC and CV (Currell & Jeukendrup, 2008). Previous studies have shown that most CODs tests yield ICCs above 0.75 (Fernandez-Fernandez et al., 2022). However, test complexity appears to substantially influence reliability. For example, in light-signal RA tests, elite futsal players demonstrated markedly lower ICC values when dribbling (0.60) compared with simple foot-tap tasks (0.77–0.83). Similarly, rugby players performing human-signal RA tests with deceptive actions showed an ICC as low as 0.31 for reaction time (Serpell, Ford & Young, 2010).

Moreover, the reliability of RA tests based on human-signal protocols requires strict control of tester timing. Evidence from soccer players showed that the CV% between two RA trials was only 5% yet tester timing correlated significantly with total test duration (*r* = 0.37, *p* = 0.04) (Altmann et al., 2022). Collectively, these findings highlight that human-signal protocols, while ecologically appealing, may be more vulnerable to reduced reliability due to operational complexity and participant familiarity compared with light-signal approaches.

This study demonstrated that the B-RAT exhibited good relative reliability among badminton players, supporting its capacity to consistently capture reactive agility in both male and female athletes. Furthermore, absolute reliability indices indicated low measurement error, enabling the detection of subtle performance differences. Several methodological features likely contributed to this robustness.

First, the B-RAT showed high ecological validity by incorporating rapid decelerations and multidirectional stepping patterns that closely mimic competitive play while remaining simple to execute. Second, standardized procedures, including uniform warm-up, equipment calibration, and movement criteria, minimized extraneous variability. Third, test duration was carefully aligned with the temporal demands of badminton rallies, which limited confounding influences. Finally, the homogeneity of the sample, as all participants were highly trained athletes with at least eight years of training experience, reduced inter-individual variability.

Notably, while the light-signal B-RAT is reliable and standardized, its perception–action coupling remains lower than that of opponent-based cues. Badminton athletes rely on opponents’ movements to anticipate and make decisions, which cannot be fully replicated by light signals. Future iterations could incorporate video simulations or opponent-triggered mechanisms to enhance the B-RAT ecological validity.

### Content validity

Performance protocols can be evaluated through three types of validity: content, criterion, and construct validity (Currell & Jeukendrup, 2008). The B-RAT’s content validity was systematically evaluated through expert review, yielding an overall S-CVI of 0.93 and item-level I-CVIs above 0.78. Specifically, the I-CVIs for definition, sport-specific cognitive component, angles of CODs, and court condition were 1.0. The sport-specific technique dimension was 0.92. Test distance, number of CODs, and task representativeness were 0.83, all exceeding the conventional 67% agreement threshold (Robertson et al., 2017), indicating that experts agreed the B-RAT captures core characteristics of badminton-specific agility.

Regarding test design, the six-target B-RAT was structured to reflect the spatiotemporal demands of badminton matches. Athletes typically start from the court center and return after each shot; therefore, single-movement distances were limited to ≤ 4 m, matching actual movement patterns. COD angles were set at 45°, 135°, and 180°, representing common abrupt stops and turns in match play. The total running distance was restricted to ≤36 m, corresponding to typical rally duration of 6–13 s and energy supply primarily from phosphagen and glycolytic systems.

Additionally, randomized visual signal stimuli simulated the rapidly changing external information in real matches, allowing B-RAT to evaluate not only movement execution and directional changes but also reaction speed and decision-making. Overall, B-RAT integrates short movement distances, multi-directional footwork, abrupt starts and stops, and cognitive stimuli, providing a reliable and practical tool for assessing badminton-specific agility.

### Criterion validity

Criterion validity includes concurrent and predictive validity (Currell & Jeukendrup, 2008). Given the absence of a gold-standard test for agility in badminton, this study focused on evaluating the predictive validity of the B-RAT. B-RAT performance was significantly positively correlated with simulated match ranking in both male (*r* = 0.65) and female (*r* = 0.76) singles players. These findings align with those of Tiwari, Rai & Srinet (2011) who reported a very large association between agility and competitive performance in Indian badminton players (*r* = −0.83), highlighting agility as a key determinant of match outcomes. Jeyaraman, District & Nadu (2012) demonstrated that, among anthropometric, physical, and physiological variables, performance in the SEMO CODs test was the best predictor of match outcomes in collegiate male badminton players (*r* = 0.74). Moreover, Paul, Gabbett & Nassis (2016) emphasized the influence of agility on performance in high-intensity, fast-reacting sports such as soccer and basketball.

The strong relationship between B-RAT performance and match ranking can be attributed to the alignment of agility with badminton’s performance-determining principles: speed, decisiveness, accuracy, and adaptability. Speed constitutes the core determinant, while decisiveness, accuracy, and adaptability function synergistically to reflect athletes’ technical and tactical execution (Chunlei, 2016). Athletes with superior agility minimize abrupt stops and starts, reduce movement distances, constrain opponents’ offensive opportunities, and enhance tactical efficiency. Additionally, they can anticipate shuttlecock trajectories within milliseconds, seize the initiative for subsequent strokes, and accumulate advantages through rapid directional changes. Consequently, agility, as a fundamental physical attribute, substantially influences singles badminton performance.

### Construct validity

Construct validity includes convergent and discriminant validity, with the latter can be measured by comparing two different groups of subjects with different abilities (Currell & Jeukendrup, 2008). In line with the expert–novice paradigm, this study examined differences in B-RAT performance between athletes of higher and lower competitive levels. Previous studies have consistently demonstrated that elite athletes outperform lower-level counterparts in RA tests employing sport-specific techniques, light-signal stimuli, or human-signal cues, with inter-group differences ranging from 4.60% to 13.32%, 4.60% to 12.74%, and 3.03% to 18.24%, respectively (Ye et al., 2025). Consistently, this study revealed significant differences in B-RAT performance between male and female athletes of differing competitive levels. Moreover, regression analyses by Jeyaraman, District & Nadu (2012) indicated that RA accounted for 46%–58% of the variance in competitive performance, further supporting the capacity of B-RAT to identify athletes’ skill hierarchies.

The high discriminant validity of B-RAT derives from its precise assessment of key sport-specific components. First, decision-making capacity varies substantially across skill levels. Badminton, as a high-intensity sport, requires rapid reactions and accurate decisions; elite athletes execute complex movements with greater efficiency, whereas lower-level athletes demonstrate slower responses and reduced movement accuracy (Scanlan, Tucker & Dalbo, 2015; Zouhal et al., 2018). Consistently, Henry et al. (2012) reported that in human-signal RA tests incorporating deceptive movements, elite athletes more effectively interpret kinematic cues and are less affected by distractors.

Second, variability in physical fitness contributes to performance differences, as athletes at different developmental stages exhibit distinct physical capacities. Finally, accumulated training effects play a critical role. Prior studies demonstrated that elite male soccer players showed larger inter-group differences in RA when assessed with dribbling-specific tasks compared with ball-contact tasks (Sekulic et al., 2019; Sekulic et al., 2021), highlighting the importance of skill specificity and training accumulation in differentiating performance levels.

Convergent validity reflects the degree of agreement among different non-gold standard tests when assessing the same construct under identical conditions (Currell & Jeukendrup, 2008). B-RAT demonstrated moderate to large correlations with commonly used badminton CODs tests (*r* = 0.48–0.64, *R*^2^ = 0.20–0.38). Comparable research in tennis players reported low correlations between RA performance and spider run (*r* = 0.39) or *T*-test CODs (*r* = 0.35) performing different movement patterns (Zhou et al., 2024). A systematic review further confirmed that COD and RA tests exhibit low to moderate correlations across different movement patterns (*r* = 0.01–0.64, *R*^2^ = 0.00–0.40), and even similar movement patterns only yielded low to moderate correlations (*r* = 0.10–0.68, *R*^2^ = 0.01–0.46) (Ye et al., 2025).

Differences in correlations between B-RAT and CODs can be primarily attributed to two factors. First, cognitive demands. Previous findings demonstrated that lateral RA (*r* = 0.52–0.66) and semicircular RA (*r* = 0.53–0.55) showed only moderate associations with CODs, whereas RA tests with higher cognitive complexity, such as integrated RA (*r* = 0.06–0.37) and frontal RA (*r* = 0.35), were non-significant (Coh et al., 2018). This suggests that the perceptual–cognitive load inherent in B-RAT reduces its shared variance with conventional CODs. Second, movement pattern specificity. B-RAT integrates RA with badminton-specific movement patterns, emphasizing rapid responses, multidirectional changes, and instantaneous decision-making. Tests closely matching B-RAT patterns, including the modified *T*-test CODs (*r* = 0.64), SEMO CODs (*r* = 0.58), and sideway CODs (*r* = 0.56), showed higher correlations, whereas less similar tests, such as the four-coner CODs (*r* = 0.48) and 505 CODs (*r* = 0.50), showed lower correlations (Altmann et al., 2021; Fiorilli et al., 2017a; Fiorilli et al., 2017b).

Thomas, Nelson & Silverman (2015) proposed that when the shared variance between two variables is below 50%, they should be considered largely independent, supporting the interpretation that CODs and RA represent distinct physical qualities. In line with this perspective, the present findings support the use of B-RAT as a standalone measure of badminton-specific agility. Given that competitive badminton performance relies heavily on instantaneous, environment-driven decisions, B-RAT provides ecologically valid and practically relevant information beyond traditional CODs tests, with direct implications for agility monitoring and targeted training prescription.

### Feasibility

The feasibility of a performance test is a prerequisite for its consistent application and broader use (Chiwaridzo et al., 2018; Robertson et al., 2017). Using a priority-based evaluation framework, this study assessed the feasibility of the B-RAT and obtained an average total score of 44.17, exceeding the predefined feasibility threshold of 35 and indicating satisfactory overall practicality. Among high-priority criteria, test procedure received the highest rating, suggesting that the testing protocol is clearly defined and easy to administer, which may improve testing efficiency and reduce operator-related variability. Equipment needed were rated slightly lower, reflecting a reliance on electronic devices; however, these requirements remain acceptable within professional and semi-professional training settings. Equipment cost showed comparatively lower scores, indicating that the initial investment in electronic response systems may be a limiting factor for implementation in resource-constrained environments.

Scores for medium-priority criteria were consistently high, indicating that the B-RAT can be completed within a reasonable time, requires minimal personnel, and allows for straightforward scoring and interpretation. Low-priority criteria also demonstrated favorable ratings, particularly for safety, while age specificity and logical acceptability were rated slightly lower. This suggests that the B-RAT is well suited for high-level athlete populations, although further research is needed to confirm its applicability in other groups. Overall, the findings indicate that the B-RAT achieves a practical balance between measurement rigor and operational feasibility, supporting its potential utility in applied sports performance settings.

### Limitations and future directions

This study has several limitations. First, the participants were primarily adolescent high-level athletes, which may limit the generalizability of the findings to adult elite players and recreational populations. Second, all participants completed all agility tests on the same day in a fixed order, which may have introduced fatigue or order effects, potentially influencing performance outcomes. Criterion validity was assessed using simulated match rankings and was based on a small sample of singles players, which may have affected the stability of the correlation coefficients and limited the interpretability of the results across different competitive formats. Future research should expand the sample to include athletes of various ages and competitive levels, implement multi-day testing protocols to reduce fatigue effects, and incorporate real match data with longitudinal tracking to improve the ecological validity and reliability of the B-RAT outcomes.

## Conclusions

This study found that the B-RAT exhibits high test–retest reliability, content validity, and construct validity, and can effectively distinguish badminton players across different competitive levels. The test further demonstrates its advantages in terms of simplicity of administration, reasonable duration, and cost-effectiveness, supporting its strong overall feasibility and potential for wide application. Coaches can use the B-RAT to assess athletic performance before and after training interventions, inform individualized training loads and content optimization, and support long-term monitoring of athlete development or identification of high-potential talent.

##  Supplemental Information

10.7717/peerj.20972/supp-1 Supplemental Information 1 Expert Questionnaire on Content Validity Evaluation

10.7717/peerj.20972/supp-2 Supplemental Information 2 Expert Questionnaire for Feasibility Evaluation

10.7717/peerj.20972/supp-3 Supplemental Information 3 Raw data

## Additional Information and Declarations

### Data Availability

The following information was supplied regarding data availability: The raw data is available in the Supplemental File.
