# Supplementary material for: Reliability, validity, and feasibility of a method for assessing sport-specific reactive agility in badminton players
Source: PeerJ. 2026 Mar 23;14:e20972. doi: 10.7717/peerj.20972 (PMC13020436; doi:10.7717/peerj.20972)
Supplement: Supplemental Information 1 [file peerj-14-20972-s001.docx]

# Appendix I: Expert Questionnaire on Content Validity Evaluation

## I. Expert Background Information

Name: ________________________ Age: ____________________________________

Research Area: _________________ Professional Title: _________________________

Affiliation: ____________________ Years of Coaching/Teaching: _____________

## II. General Information on the Newly Designed Badminton-Specific Reactive Agility Test

The badminton-specific reactive agility test developed in this study is based on the Reaction X system (Reaction X, China). Compared with expensive devices such as Fitlight and Smartspeed, the cost of six Reaction X lights is approximately 1,400 RMB, while offering advantages including long standby time, convenient charging, portability, and rapid assembly.

In the B-RAT, six shuttlecocks serving as touch targets form a 6.2 m × 5.18 m rectangle. They are positioned at the singles sideline 0.5 m from the net, at the intersections of the baseline with the singles sidelines, and at the midpoints of the rectangle’s longer sides. A height-adjustable tripod (1.2 m) is placed 0.5 m in front of the rectangle’s center point. The reaction lights are attached with Velcro to a panel on the tripod, corresponding to six directions: upper-right, right, lower-right, lower-left, left, and upper-left. The Reaction X software, downloaded to an iPad (Apple, USA), controls the lights via Bluetooth. The test is configured with sequence mode, 0.5 s delay, logical randomization, sound-on light cues, sound-on touch cues, short-distance sensor mode, and one cycle.

**Schematic diagram of badminton-specific reactive agility test**

During testing, the athlete stands at the center of the rectangle in a ready stance with feet parallel. When a reaction light flashes, the athlete sprints quickly to the corresponding shuttlecock, touches it, then immediately returns to the center and touches the flashing reaction light. After touching all six shuttlecocks and their corresponding lights in random directions without repetition, the test ends. Throughout the procedure, athletes must use badminton-specific footwork and touch both the shuttlecock and the light with their racket hand. The light sequence is randomized, and the software automatically records split times for each direction and the total completion time.

## III. Dimensions for Content Validity Evaluation

Experts are required to rate each item of the content validity scale according to its relevance to agility performance in actual badminton competition contexts, using the 4-point scale proposed by Davis (1992). Please mark “√” in the corresponding “.” The scoring criteria are as follows:

1 = Not relevant: Does not meet the evaluation criteria; unable to reflect actual agility performance.

2 = Somewhat relevant: Partially meets the criteria but with large discrepancies from practical application.

3 = Quite relevant: Meets the evaluation criteria and has certain applicability.

4 = Highly relevant: Strongly meets the evaluation criteria and is applicable to real competition contexts.

**1. Definition of Agility:** Agility refers to the ability of the body to rapidly change speed or direction in response to external stimuli. Please evaluate whether the newly designed badminton-specific agility test conforms to this definition.

1 = Not relevant: Does not simulate or reflect athletes’ ability to react quickly and change speed/direction during competition; fails to capture the core requirement of agility.

2 = Somewhat relevant: Partially involves speed or direction changes, but the test does not fully reflect the reactive demands of competition.

3 = Quite relevant: Adequately simulates athletes’ response to external stimuli in badminton competition and partially reflects the core requirements of agility.

4 = Highly relevant: Fully reflects badminton players’ ability to rapidly change speed or direction during competition, completely consistent with the definition of agility.

**2. Sport Cognition:** Does the newly designed badminton-specific agility test encompass athletes’ ability to react quickly to external stimuli (i.e., decision-making and judgment)?

1 = Not relevant: Does not involve reaction speed or decision-making ability; cannot assess athletes’ sport cognition.

2 = Somewhat relevant: Includes some requirements to respond to external stimuli but lacks decision-making and judgment components; insufficient for evaluating sport cognition.

3 = Quite relevant: Adequately incorporates rapid response elements to external stimuli, allowing for assessment of sport cognition.

4 = Highly relevant: Accurately simulates athletes’ ability to make quick decisions and responses to complex signals in competition, fully meeting the demands of sport cognition.

**3. Sport-Specific Techniques:** Does the newly designed badminton-specific agility test incorporate key technical features of badminton, such as rapid footwork, reactive strokes, and positional changes on the court?

1 = Not relevant: Does not involve badminton-specific techniques; fails to reflect athletes’ technical performance in competition.

2 = Somewhat relevant: Includes some badminton-specific movements, but the technical features do not adequately reflect the actual demands of competition; test design remains simplistic.

3 = Quite relevant: Adequately integrates some key technical features of badminton, such as footwork and positional adjustments, and reflects certain technical abilities required in competition.

4 = Highly relevant: Fully incorporates badminton-specific techniques, including reactive strokes, rapid movement, and court repositioning, completely reflecting the technical features of real competition.

**4. Test Distance:** Does the movement distance in the newly designed badminton-specific agility test reflect the actual movement demands of a badminton match?

1 = Not relevant: The test distance differs greatly from the actual movement distance in badminton.

2 = Somewhat relevant: Part of the test distance meets the movement requirements of badminton, but the overall design is not entirely appropriate.

3 = Quite relevant: The test distance adequately simulates the movement range in badminton matches.

4 = Highly relevant: The test distance fully matches the actual movement demands of badminton matches.

**5. Number of Direction Changes:** Does the number of direction changes in the newly designed agility test simulate actual badminton match situations?

1 = Not relevant: The number of changes of direction in the test is far from that required in actual badminton.

2 = Somewhat relevant: Partly reflects the demands of badminton, but simulation effect is weak.

3 = Quite relevant: Reasonably simulates the actual movement demands of badminton.

4 = Highly relevant: Closely matches the actual demands of matches and fully simulates complex directional changes.

6. **Angle of Direction Changes**: Do the directional change angles in the test meet the demands of badminton?

1 = Not relevant: The angles are overly simplified and fail to reflect the complexity of badminton match demands.

2 = Somewhat relevant: Some angles correspond to match requirements but do not fully capture the complexity.

3 = Quite relevant: Angles reasonably simulate the actual directional demands of badminton matches.

4 = Highly relevant: Angles are well designed and fully reflect the complex demands of badminton matches.

**7. Test Time Arrangement:** Does the time setting of the test reflect the temporal demands of badminton rallies?

1 = Not relevant: Test duration is oversimplified, focusing only on repetitions without reflecting rally time demands.

2 = Somewhat relevant: Records only partial time data, failing to comprehensively reflect rally demands.

3 = Quite relevant: Adequately records athletes’ overall performance and simulates rally time demands.

4 = Highly relevant: Fully incorporates time design, including split times, total duration, and repetitions, simulating rally time demands comprehensively.

8. Test Venue: Is the test venue consistent with the actual badminton competition environment?

1 = Not relevant: Conducted in a laboratory setting, failing to simulate real match conditions.

2 = Somewhat relevant: Venue conditions differ significantly from actual match settings.

3 = Quite relevant: Venue conditions reasonably simulate the competition environment.

4 = Highly relevant: Conducted entirely on a standard badminton court, highly consistent with match conditions.

## IV. Open-Ended Question (Feedback)

In your opinion, what aspects of the newly designed badminton-specific reactive agility test could be further optimized to better reflect the actual agility demands of competitive badminton?
