# Supplementary material for: Reliability, validity, and feasibility of a method for assessing sport-specific reactive agility in badminton players
Source: PeerJ. 2026 Mar 23;14:e20972. doi: 10.7717/peerj.20972 (PMC13020436; doi:10.7717/peerj.20972)
Supplement: Supplemental Information 2 [file peerj-14-20972-s002.docx]

# Appendix II: Expert Questionnaire for Feasibility Evaluation

## I. Expert Background Information

Name: ________________________ Age: ____________________________________

Research Area: _________________ Professional Title: _________________________

Affiliation: ____________________ Years of Coaching/Teaching: _____________

## II. General Information on the Newly Designed Badminton-Specific Reactive Agility Test

The badminton-specific reactive agility test developed in this study is based on the Reaction X system (Reaction X, China). Compared with expensive devices such as Fitlight and Smartspeed, the cost of six Reaction X lights is approximately 1,400 RMB, while offering advantages including long standby time, convenient charging, portability, and rapid assembly.

In the B-RAT, six shuttlecocks serving as touch targets form a 6.2 m × 5.18 m rectangle. They are positioned at the singles sideline 0.5 m from the net, at the intersections of the baseline with the singles sidelines, and at the midpoints of the rectangle’s longer sides. A height-adjustable tripod (1.2 m) is placed 0.5 m in front of the rectangle’s center point. The reaction lights are attached with Velcro to a panel on the tripod, corresponding to six directions: upper-right, right, lower-right, lower-left, left, and upper-left. The Reaction X software, downloaded to an iPad (Apple, USA), controls the lights via Bluetooth. The test is configured with sequence mode, 0.5 s delay, logical randomization, sound-on light cues, sound-on touch cues, short-distance sensor mode, and one cycle.

**Schematic diagram of badminton-specific reactive agility test**

During testing, the athlete stands at the center of the rectangle in a ready stance with feet parallel. When a reaction light flashes, the athlete sprints quickly to the corresponding shuttlecock, touches it, then immediately returns to the center and touches the flashing reaction light. After touching all six shuttlecocks and their corresponding lights in random directions without repetition, the test ends. Throughout the procedure, athletes must use badminton-specific footwork and touch both the shuttlecock and the light with their racket hand. The light sequence is randomized, and the software automatically records split times for each direction and the total completion time.

## III. Dimensions for Feasibility Evaluation

Experts were asked to rate each category based on practical feasibility using a 3-point scale (0 = Not feasible, 1 = Somewhat feasible, 2 = Feasible) and mark “√” in the “” column

**1. Equipment Requirements**

 0 = Not feasible: Required equipment is difficult to obtain, complex to operate, and hard to maintain.

 1 = Somewhat feasible: Equipment is obtainable but may be difficult to access or operate.

 2 = Feasible: Equipment is easy to obtain and operate.

**2. Test Procedure**

 0 = Not feasible: Procedure is complex, hard to understand or execute, requiring excessive time and steps.

 1 = Somewhat feasible: Procedure is generally clear but may have complex steps or need optimization.

 2 = Feasible: Procedure is clear, concise, and easy to execute; steps are logical and understandable.

**3. Practicality Improvements**

 0 = Not feasible: Major issues exist in practical application, cannot be implemented effectively, or lacks room for improvement.

 1 = Somewhat feasible: Some practical issues exist but can be improved for better usability.

 2 = Feasible: Highly practical, can be applied smoothly in real settings without major modifications.

**4. Equipment Cost**

 0 = Not feasible: Costs are too high, exceeding budget and difficult to afford.

 1 = Somewhat feasible: Costs are moderate but may strain the budget.

 2 = Feasible: Costs are reasonable, within budget, and easily affordable.

**5. Test Duration**

 0 = Not feasible: Duration is too long, disrupting training or causing inconvenience.

 1 = Somewhat feasible: Duration is somewhat long but acceptable, with limited impact on training or preparation.

 2 = Feasible: Duration is appropriate and does not significantly affect training or preparation.

**6. Number of Test Personnel**

 0 = Not feasible: Requires too many personnel, making coordination difficult and operations complex.

 1 = Somewhat feasible: Personnel number is moderate but may need extra coordination.

 2 = Feasible: Personnel number is reasonable, easy to coordinate, and simple to manage.

**7. Difficulty of Scoring and Interpretation**

 0 = Not feasible: Scoring and interpretation are complex, hard to understand, and require specialized training.

 1 = Somewhat feasible: Scoring and interpretation are generally manageable but may require some training.

 2 = Feasible: Scoring and interpretation are simple and easy to understand without extra training.

**8. Age Specificity**

 0 = Not feasible: Test is not suitable for different age groups and targets only a specific age range.

 1 = Somewhat feasible: Test suits certain ages but may need adjustment for all age groups.

 2 = Feasible: Test is suitable for all age groups without modification.

**9. Logical Acceptability**

 0 = Not feasible: Test design logic is inconsistent with athletes’ actual needs, difficult to accept.

 1 = Somewhat feasible: Logic is generally acceptable but could be improved for better acceptance.

 2 = Feasible: Logic fully aligns with athletes’ needs and is easily accepted.

**10. Safety**

 0 = Not feasible: Test has clear safety risks that may harm athletes.

 1 = Somewhat feasible: Generally safe but minor risks exist and should be noted.

 2 = Feasible: Test design ensures high safety and minimal risk.

**11. Open-Ended Feedback on Feasibility**

Experts were invited to provide suggestions on areas for improvement to further enhance the practical applicability of the newly designed badminton-specific agility te
